# INCLUSION OF ETHNICALLY DIVERSE POPULATIONS IN CLINICAL TRIALS OF HEALTHSPAN: IMPLICATIONS FROM THE SALSA STUDY

**DOI:** 10.1093/geroni/igac059.1853

**Published:** 2022-12-20

**Authors:** Sara Espinoza, Tiffany Cortes, Helen Hazuda

**Affiliations:** UT Health San Antonio, San Antonio, Texas, United States; UT Health San Antonio, San Antonio, Texas, United States; UT Health San Antonio, San Antonio, Texas, United States

## Abstract

**Background:**

Geroscience-guided clinical trials focused on healthspan may seek to enroll older adults initially free of chronic diseases and disability. Here we examine healthspan in the San Antonio Longitudinal Study of Aging (SALSA), a cohort of 749 community-dwelling older (65+ years) Mexican Americans (MA) and European Americans (EA), and describe prevalence and characteristics associated with poor healthspan.

**Methods:**

Poor healthspan was defined at the SALSA baseline exam as presence of any one of: 1) chronic disease (diabetes, myocardial infarction, congestive heart failure, stroke, chronic obstructive pulmonary disease, or cancer); 2) dependence in basic or instrumental activities of daily living; or 3) mini mental state exam score < 18. Frailty was defined by Fried phenotype criteria. The association of poor healthspan with age, sex, ethnic group, socioeconomic status (SES), and frailty was assessed using chi-square or t-tests.

**Results:**

544 (72.6%) participants met criteria for poor healthspan, which was associated with older age (69.6 ±3.4 vs. 69.3 ±3.4, p< 0.05), male sex (77.3% vs. 69.2%, p< 0.05), MA ethnicity (77.4% vs. 67.3%, p< 0.05), lower income (11.3 ±3.1 vs. 12.2 ±2.9, p< 0.001 and education (10.4 ±4.6 vs. 12.7 ±3.2, < 0.0001), and frailty (95.5% vs. 4.6%, p< 0.001).

**Conclusion:**

Poor healthspan was highly prevalent (>70%) in SALSA and associated with MA ethnicity, low SES, and frailty. Geroscience-guided clinical trials of potential interventions to improve healthspan by preventing chronic diseases and disability may under-represent individuals of ethnic minority background and lower SES and, thereby, jeopardize generalizability of the findings to the broader population.

